# Sexual dimorphism in prostacyclin‐mimetic responses within rat mesenteric arteries: A novel role for K_V_7.1 in shaping IP receptor‐mediated relaxation

**DOI:** 10.1111/bph.15722

**Published:** 2022-01-21

**Authors:** Samuel N. Baldwin, Elizabeth A. Forrester, Lauren McEwan, Iain A. Greenwood

**Affiliations:** ^1^ Molecular and Clinical Sciences Research Institute St. George's University London UK

**Keywords:** GPCR, Iloprost, Kv7, MRE‐269, Prostacyclin, Sex, Vascular

## Abstract

**Background and Purpose:**

Prostacyclin mimetics express potent vasoactive effects via prostanoid receptors that are not unequivocally defined, as to date no study has considered sex as a factor. The aim of this study was to determine the contribution of IP and EP_3_ prostanoid receptors to prostacyclin mimetic iloprost‐mediated responses, whether K_V_7.1–5 channels represent downstream targets of selective prostacyclin‐IP‐receptor agonist MRE‐269 and the impact of the oestrus cycle on vascular reactivity.

**Experimental Approach:**

Within second‐order mesenteric arteries from male and female Wistar rats, we determined (1) relative mRNA transcripts for EP_1–4_ (*Ptger*_*1–4*_), IP (*Ptgi*) and TXA_2_ (*Tbxa*) prostanoid receptors via RT‐qPCR; (2) the effect of iloprost, MRE‐269, isoprenaline and ML277 on precontracted arterial tone in the presence of inhibitors of prostanoid receptors, potassium channels and the molecular interference of K_V_7.1 via wire‐myograph; (3) oestrus cycle stage via histological changes in cervical cell preparations.

**Key Results:**

Iloprost evoked a biphasic response in male mesenteric arteries, at concentrations ≤100 nmol·L^−1^ relaxing, then contracting the vessel at concentration ≥300 nmol·L^−1^, a process attributed to IP and EP_3_ receptors respectively. Secondary contraction was absent in the females, which was associated with a reduction in *Ptger3*. Pharmacological inhibition and molecular interference of K_V_7.1 significantly attenuated relaxations produced by the selective IP receptor agonist MRE‐269 in male and female Wistar in dioestrus/metoestrus, but not pro‐oestrus/oestrus.

**Conclusions and Implications:**

Stark sexual dimorphisms in iloprost‐mediated vasoactive responses are present within mesenteric arteries. K_V_7.1 is implicated in IP receptor‐mediated vasorelaxation and is impaired by the oestrus cycle.

**What is already known**

- The prostacyclin analogue iloprost evokes vasoactive responses through a myriad of receptors.
- K_V_7 channels are targets of endogenous vasoactive signalling cascades.

**What does this study add**

- Iloprost evoked biphasic relaxant‐contrail responses in males, which is absent in females
- K_V_7.1 inhibition impairs MRE‐269 mesenteric arteries relaxation which is affected by the oestrus cycle.

**What is the clinical significance**

- Sex must be considered as a factor when considering prostacyclin mimetics as a therapeutic tool.

## 1 INTRODUCTION

Prostacyclin (PGI_2_), a product cyclooxygenase‐1 (COX‐1) and cyclooxygenase‐2 (COX‐2) metabolism of arachidonic acid, has anti‐thrombotic, anti‐inflammatory and potent vasodilatory properties attributed to the activation of G_s_‐coupled prostanoid (IP) receptor signalling. PGI_2_ and stable analogues, including iloprost, however bind to a plethora of G‐protein coupled receptors (GPCR) including EP _1_ _,_ _2_ _,_ _3_ _,_ _4_ , IP, TP, FP and DP_1_ (Katusic et al., 2012), though with different affinities. For instance, in human and rat pulmonary arteries, iloprost binds to IP/EP_1_ with high affinity, FP > EP_3/4_ with moderate affinity and DP_1_ > EP_2_ > TP with low affinity (Whittle et al., 2012). As such, the potential effect of PGI_2_/iloprost within the vasculature includes both EP_1/3_/FP prostanoid receptor‐evoked contraction (Kobayashi et al., 2011; Orie & Clapp, 2011; Tang et al., 2008) and EP_4_/IP prostanoid receptor‐mediated relaxation (e.g. Dumas et al., 1997; Lombard et al., 1999; Schubert et al., 1996, 1997). These diverse responses to prostacyclin mimetics remain largely uncharacterized and, moreover, few studies have considered sex as a factor when characterizing prostacyclin‐memetic mediated vascular responses.

Previously, large conductance calcium activated potassium channels (K_Ca_1.1/BK_Ca_ ; Schubert et al., 1997), ATP‐sensitive potassium channels (K_ATP_ ; Lombard et al., 1999; Schubert et al., 1997) and inwardly rectifying potassium channels (K_IR_ ; Orie et al., 2006) have been identified as the downstream targets of IP receptor‐mediated vasorelaxation. Voltage‐gated potassium channels encoded by *KCNQ1‐5* genes (termed K_V_7.1‐5 channels) are voltage‐gated potassium channels with a negative threshold for activation that have well‐identified roles in maintaining resting excitability in neurones, cardiac myocytes, epithelia and smooth muscle cells (Barrese et al., 2018). Of the five subtypes genes, *Kcnq1* , *4* and *5* are robustly expressed in arterial smooth muscle (Barrese et al., 2020; Mackie et al., 2008; Yeung et al., 2007) and blockers of the expressed channels elicit contraction or enhanced vasoconstrictor response (e.g. Mackie et al., 2008; Yeung et al., 2007). K_V_7 channels are also key functional components of vasorelaxations generated by several agonists of G_s_‐linked receptors including β‐adrenoceptors (Chadha, Zunke, Zhu, et al., 2012; Stott et al., 2016), calcitonin gene‐related peptide receptors (Chadha et al., 2014; Stott et al., 2018) and adenosine receptors (Khanamiri et al., 2013). Additionally, novel findings implicate sexual dimorphisms in channel physiology (Abbott & Jepps, 2016; Alzamora et al., 2011) and pathophysiology (Berg, 2018).

As the contribution of K_V_7 channels to prostanoid receptor‐mediated relaxations is unknown, we sought to determine whether K_V_7 channels were involved with prostacyclin mimetic‐mediated responses in rat mesenteric arteries from aged‐matched male and female rats. This artery was chosen because (1), K_V_7 expression has been established (Jepps et al., 2011, 2015; Mackie et al., 2008), (2) K_V_7 activators are effective relaxants (Jepps et al., 2014), (3) a role for K_V_7 channels in G_αs_‐linked responses has been identified (e.g. Lindman et al., 2018; Stott et al., 2016, 2018) and (4), endothelium dependent production of PGI_2_ mediates concomitant IP receptor‐mediated relaxation (Liu et al., 2012) and TP/EP_3_‐mediated contraction (Liu et al., 2012, 2017). Moreover, in line with Docherty et al. (2019), we investigated possible sex difference as nothing is known about the impact of sex on prostanoid‐mediated vascular responses. To circumvent the short half‐life of prostacyclin, we characterized the contribution of EP_3_ and IP receptors to responses mediated by its stable analogue iloprost and defined the contribution of K_V_7 channels to IP receptor‐mediated vasorelaxation using a selective IP receptor agonist, MRE‐269. Our data demonstrate a striking sex‐dependent difference in response to iloprost and a role for K_V_7.1 channels in shaping IP receptor‐evoked vasorelaxation within rat mesenteric arteries, which is oestrus cycle sensitive.

## 2 METHODS

### 2.1 Animal models

Animal studies are reported in compliance with the ARRIVE guidelines (Percie du Sert et al., 2020) and with the recommendations made by the *British Journal of Pharmacology* (Lilley et al., 2020). Experiments were performed on arteries from male and female Wistar rats (RRID:RGD_734476, Charles River, Margate, UK) ages 11–14 weeks (200–350 g) housed at the Biological Research Facility, St George's, London, UK. A maximum of 5 rats were housed in NKP cages with free access to water and food (RM1; Dietex Inter‐national, UK) with a 12‐h light/dark cycle and constant temperature and humidity (21 ± 1°C; 50% ± 10% humidity) in accordance with the Animal (Scientific Procedures) Act 1986. Animals were kept in LSB Aspen woodchip bedding. Animals were killed by cervical dislocation with secondary confirmation via cessation of the circulation by femoral artery severance in accordance with Schedule 1 of the ASPA 1986.

For the following investigations, second‐order mesenteric arteries were used, identified as the second bifurcation of the superior mesenteric artery. Arteries were dissected, cleaned of fat and adherent tissue and stored on ice in physiological salt solution (PSS) of the following composition (mmol·L^−1^): 119 NaCl, 4.5 KCl, 1.17 MgSO_4_·7H_2_0, 1.18 NaH_2_PO_4_, 25 NaHCO_3_, 5 glucose, 1.25 CaCl_2_.

### 2.2 Oestrus cycle stage determination

After killing, 50 μl of PSS was inserted into the vaginal canal via a 2‐ to 200‐μl pipette tip and flushed four to six times to liberate cells from the surface of the cervix and then stored on ice; 25 μl of the subsequent cell suspension was mounted on a glass slide and examined under light microscopy (×10 to ×20 magnification). Previously described changes in cervical cell histology allowed for the determination of oestrus cycle stage (Cora et al., 2015) as either (in order of the 4–5 day cycle) pro‐oestrus, oestrus, metoestrous or dioestrus. Cycle stage determination was performed post‐experiment during functional investigation as a means of blinding; this was not possible during molecular techniques.

### 2.3 Wire myography

For functional investigations, ~2‐mm arterial segments were mounted on a 40‐μm tungsten steel wire within a myograph chamber (Danish Myo Technology, Arhus, Denmark) containing 5 ml of PSS (composition described above) oxygenated with 95% O_2_ and 5% CO_2_ at 37°C. Vessels then underwent a passive force normalization process to achieve an internal luminal circumference at a transmural pressure of 100 mmHg (13.3 kPa) to standardize pre‐experimental conditions (Mulvany, 1977). Force generated was first amplified by a PowerLab (ADInstruments, Oxford, UK) and then recorded via LabChart software (RRID:SCR_017551; ADInstruments, Oxford, UK). Vessels were then challenged with isotonic high K^+^ physiological salt solution (K^+^PSS) of the following composition (mmol·L^−1^): 63.5 NaCl, 60 KCl, 1.17 MgSO_4_·7H_2_0, 1.18 NaH_2_PO_4_, 25 NaHCO_3_, 5 glucose, 1.25 CaCl_2_, to determine viability. Vessels were then constricted with 10 μmol·L^−1^ methoxamine, an α_1_‐adrenoreceptor agonist, and endothelial cell integrity was determined via vasorelaxation in response to 10 μmol·L^−1^ of the synthetic acetylcholine analogue, carbachol, prior to investigation. Subsequently, vessels were preconstricted with 300 nmol·L^−1^ thromboxane A_2_ mimetic U46619. Following which single dose responses or cumulative concentration effect curve were generated in response to either prostacyclin mimetic iloprost (0.001–3 μmol·L^−1^), IP receptor agonists selexipag (0.03–3 μmol·L^−1^)/MRE‐269 (0.01–1/0.01–3 μmol·L^−1^), β‐adrenoreceptor agonist isoprenaline (0.03–3 μmol·L^−1^) or K_V_7.1 activator ML277. Vessels were preincubated in the presence or absence of a combination of solvent control dimethyl sulphoxide (DMSO) or antagonists of the following: ‐ IP prostanoid receptor‐CAY10441 (RO1138452; 100 nmol·L^−1^); EP_3_ prostanoid receptor‐L‐798,106 (300 nmol·L^−1^); pan‐K_V_7 channel, linopirdine (10 μmol·L^−1^); K_V_7.1, HMR‐1556 (10 μmol·L^−1^); adenylate cyclase ,SQ22,562 (10 μmol·L^−1^); PKA, Rp‐8‐Br‐cAMP/KT5720 (1 μmol·L^−1^); EPAC (100 nmol·L^−1^); G_β**γ**_, M119K (10 μmol·L^−1^); BK_Ca_/ K_Ca_1.1, iberiotoxin (100 nmol·L^−1^); K_ATP_, glibenclamide (1 μmol·L^−1^) for a period of 10 min.

### 2.4 Reverse transcription quantitative polymerase chain reaction

mRNA from whole mesenteric arteries was extracted using Monarch Total RNA Miniprep Kit (New England BioLabs, Ipswich, Massachusetts, USA) and reverse transcribed via LunaScript RT SuperMix Kit (New England BioLabs, Ipswich, Massachusetts, USA). Quantitative analysis of relative gene expression was determined via CFX‐96 Real‐Time PCR Detection System (RRID:SCR_018064, BioRad, Hertfordshire, UK). Samples were run in BrightWhite qPCR plate (Primer Design, Camberley, UK) in combination with PrecisionPLUS qPCR Master Mix (Primer Design, Camberley, UK), 300 nmol·L^−1^ of gene specific target primer (Thermofisher scientific, Waltham, Massachusetts, USA) and 10‐ng cDNA as per manufacturers instruction. Quantification cycle (Cq) was determined via Bio‐Rad CFX96 Manager 3.0. Cq values are expressed as normalized values to appropriate, stable, housekeeper genes (2^−ΔCq^) calnexin (*Canx*) and cytochrome C1 (*Cyc1*) chosen for their stable, and similar Cq values. Housekeeper genes were acquired from Primer Design (Camberley, UK); as such, for proprietary reasons, the sequences are not disclosed. See Table 1 for a list of primers used in the following investigation.

**TABLE 1:** RT‐qPCR primer sequences

Gene name	(+) Forward primer sequence 3′‐5′ (−) Reverse primer sequence 5′‐3′	Gene accession number	Amplicon	Concentration
EP_1_ (*Ptger1*)	(+) AGTTCGAACGTTGGTCACGA (−) TAAGGTTGCAGCATTGTGCG	NM_001278475.1	112	300 nmol·L^−1^
EP_2_ (*Ptger2*)	(+) TATGCTCCCTGCCTTTCACAA (−) GGAGGTCCCACTTTTCCTTT	NM_031088.2	72	300 nmol·L^−1^
EP_3_ (*Ptger3*)	(+) GTGCAATTCCTTCCTAATCGCC (−) TCAGGTTGTTCATCATCTGGCA	NM_012704.1	122	300 nmol·L^−1^
EP_4_ (*Ptger4*)	(+) ATGAGCATTGAGCGCTACCT (−) AGATGCATAGACGGCGAAGA	NM_032076.3	102	300 nmol·L^−1^
IP (*Ptgir*)	(+) TGACACTTTCGCCTTCGCTA (−) TAGATGGCAGGCAAAGCCAA	NP_001071112.1	156	300 nmol·L^−1^
TXA2 (*Tbxa2r*)	(+) TTGACATTCCCAGGCCCAAA (−) ACGTGATAAGGGGGTCAACA	NM_017054.2	141	300 nmol·L^−1^
Clnexin (*Canx)*	N/A (Primer design, Camberley, UK)			300 nmol·L^−1^
Cytochrome C1 (*Cyc1*)	N/A (Primer design, Camberley, UK)			300 nmol·L^−1^

### 2.5 Morpholino transfection

Knockdown of K_V_7.1 in whole mesenteric arteries was performed by transfection with morpholinos that prohibit protein translation but do not affect transcript levels (see Barrese et al., 2020; Jepps et al., 2015). Either K_V_7.1 morpholino nucleotides or mismatch controls (5 μmol·L^−1^, RRID:SCR_005663, Genetools, USA) were mixed with Lipofectamine 2000 (ThermoFisher, Paisley, UK) and Opti‐MEM (Sigma, UK) and left at room temperature for 2 h. Morpholino/Lipofectamine/Opti‐MEM mixture was added to Dulbecco's modified Eagle medium (DMEM) F‐12 (Sigma, UK) containing 1% penicillin/streptomycin. Arteries were added and left for 48 h at 37°C with 5% CO_2_.

### 2.6 Immunocytochemistry

The Immuno‐related procedures used comply with the recommendations made by the *British Journal of Pharmacology* (Alexander et al., 2018). Vascular smooth muscle cells were isolated from morpholino‐transfected mesenteric arteries via incubation in isolation PSS of the following composition (mmol·L^−1^): 120 NaCl, 6 KCl, 12 glucose, 10 HEPEs and 1.2 MgCl_2_ supplemented with 1.75 mg·ml^−1^ Collagenase Type IA, 0.9 mg·ml^−1^ protease, 1 mg·ml^−1^ trypsin inhibitor and 1 mg·ml^−1^ bovine serum albumin (Sigma, UK) at 37°C for 17 min. Vessels then underwent mechanical trituration by wide bore glass pipette to liberate vascular smooth muscle cells. The subsequent cell suspension was plated onto 13‐mm coverslips in a 24‐well plate, supplemented with an equal volume of Ca^2+^ (2.5 mmol·L^−1^) containing PSS and left to attach for 1 h.

Vascular smooth muscle cells were fixed in 3% paraformaldehyde for 15 min and then stored at 4°C in PBS prior to staining. Cells were then incubated in the following: 100 mmol·L^−1^ glycine in PBS, 5 min; blocking solution (PBS containing 0.1% Triton X‐100 and 10% FBS in PBS), 45 min; primary antibody (K_V_7.1, 1:100, Rabbit, Pineda Antikörper‐Service, Germany), overnight at 4°C. The following day, cells were incubated in secondary antibody (Goat anti‐rabbit IgG [H + L] cross‐absorbed secondary antibody, Alexa Fluro™ 568, 1:100, Goat, RRID:AB_143157, ThermoFisher, UK), then mounted in Vectasheild (Sigma, P4170). Cells were imaged via Nikon A1R confocal microscope (inverted) on Ti2 chassis (Image Resource Facility, St George's University, London), and total cell fluorescence was analysed using ImageJ (RRID:SCR_003070) software.

### 2.7 Cell culture

The K_v_7.1 antibody was validated using Chinese Hamster Ovarian (CHO) cells (RRID:CVCL_0213) overexpressing KCNQ1. CHO cells were grown in DMEM/F‐12 (Sigma, UK) supplemented in 1% penicillin/streptomycin in an incubator with 5% CO_2_ at 37°C. CHO cells were incubated with either a total of 3 μg of plasmid containing *Kcnq1* (University of Copenhagen, Denmark) in a Lipofectamine 2000/Opti‐MEM mixture (*Kcnq1*‐transfected CHO), or lipofectamine/Opti‐MEM only for 24 h (nontransfected CHO). Cells were mounted onto glass coverslips, fixed and stained for K_V_7.1 as above. Antibody specificity demonstrated by positive staining for K_V_7.1 (Pineda Antikörper‐Service, Germany) in *Kcnq1*‐transfected CHO cells (Figure S1A), but not non‐transfected CHO cells (Figure S1B).

### 2.8 Materials

All drugs for the following investigation were procured from Tocris Bioscience (Oxford, UK). Excluding CAY‐10441 and MRE‐269, which were acquired from Cayman chemical (Michigan, USA), Rp‐8‐Br‐cAMP which was aquired from Sigma‐Aldrich (UK) and M119K which was provided by the National Cancer Institute Drug Development Programme. All drugs were dissolved in DMSO and final vehicle concentrations were always ≤0.01. For materials regarding morpholino transfection, immunodetection or RT‐qPCR, see relevant sections above.

### 2.9 Data and statistical analysis

All values are expressed as mean ± standard error of the mean (SEM) for no less than five independent data points, excluding measurement of total cell fluorescence during immunocytochemistry, in which 10 cells were measured per cell. For isometric tension recordings, single dose responses to iloprost are expressed as (%) change from stable tone in response to 300 nmol·L^−1^ U46619, contractions from basal tone are expressed as (%) contraction when normalized to vasoconstriction to 10 μmol·L^−1^ methoxamine and all cumulative concentration effect curves are expressed as (%) stable contraction in response to 300 nmol·L^−1^ U46619. This is to account for changes in vessel contractility. For functional experiments involving cumulative concentration effect curves, a transformed data set was generated using; X = Log(X), to reduce representative skew. Following which, either a four parametric linear regression analysis was performed using either (Log(Agonist) vs. response variable slope [four parameters Bottom/Hillslope/top/EC_50_]) using GraphPad Prism (RRID:SCR_002798, Version 9.0.0) to fit a cumulative concentration effect curve to the figure. When generating data with morpholino‐transfected arteries, the investigator was blinded, whereby a second researcher would mount the vessels and then post‐investigation reveals which arteries had been transfected with scrambled control or *Kcnq1* targeted morpholino. Blinding for the remaining investigations was impractical because researchers were working in isolation during the COVID‐19 pandemic. However, vessel segments were cut prior to mounting during isometric tension recording and invert in an Eppendorf suspended in PSS. In doing so, vessel segments either proximal or distal to the bifurcation from first‐order mesenteric arteries were randomly selected for drug treatments. For data comparing multiple groups, a paired Student's t‐test or a two‐way ANOVA was performed for comparison of mean values. If statistical significance was achieved up two‐way ANOVA (*P* < 0.5), a post hoc Bonferonni (for comparing one condition against control) test or Dunnett's (for comparing multiple conditions against control) test was also performed to generate corrected significance values. Significant values are represented as *P* < 0.05 (*/#). Sample sizes subject to statistical analysis contain at least 5 animals per group, where *n*= number of independent values. Investigations expressing groups. Investigations expressing groups of unequal numbers were gathered due to technical failure or an artefact of cycle stage determination post‐experiment during functional investigations. The data and statistical analysis comply with the recommendations of the *British Journal of Pharmacology* on experimental design and analysis in pharmacology in accordance with Curtis et al. (2018).

### 2.10 Nomenclature of targets and ligands

Key protein targets and ligands in this article are hyperlinked to corresponding entries in the IUPHAR/BPS Guide to PHARMACOLOGY http://www.guidetopharmacology.org and are permanently archived in the Concise Guide to PHARMACOLOGY 2021/22 (Alexander, Christopoulos, et al., 2021; Alexander, Mathie, et al., 2021).

## 3 RESULTS

### 3.1 Iloprost‐mediated vasoactive responses

**FIGURE 1:**
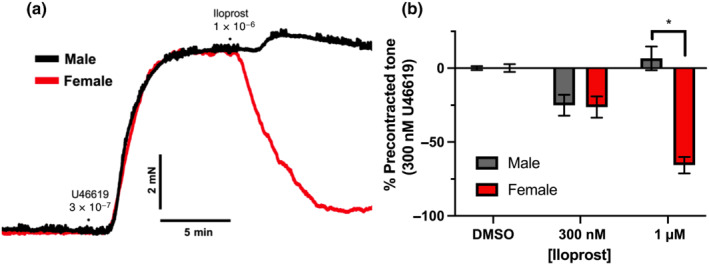
Iloprost evokes biphasic vasoactive responses within male, but not female mesenteric arteries. Representative traces of iloprost (0.3–1 μmol·L^−1^) mediated vasoactive responses on precontracted tone (300 nmol·L^−1^ U46619) within mesenteric arteries from male (a; black) or female (a; red) Wistar rats. Mean data for DMSO solvent control and iloprost‐mediated vasoactive responses on precontracted (300 nmol·L^−1^ U46619) mesenteric arteries (a; 0.3–1 μmol·L^−1^; *n* = 6–8). All values are expressed as mean ± SEM. A two‐way statistical ANOVA with a post‐hoc Bonferroni test was used to generate significance values (^*^ *P* < 0.05). *n* = number of animals used

In mesenteric arteries from male rats stably contracted by U46619 (300 nmol·L^−1^), iloprost (0.1–3 μmol·L^−1^) evoked a biphasic response, producing relaxation at lower concentrations followed by contraction at concentrations >300 nmol·L^−1^ (Figure 1a,b, black). This phenomenon was notably absent in mesenteric arteries from female rats, wherein iloprost only evoked concentration‐dependent vasorelaxation (Figure 1a,b, red). See representative traces in Figure 1a. Application of solvent control had negligible effect on established tone in mesenteric arteries from either male or female rats (Figure 1b). For concentration effect curves generated in response to iloprost on precontracted tone, see Figure S2. Prostacyclin‐mediated relaxations are conventionally mediated via activation of IP receptors, while EP_3_ receptors have been implicated in contractile responses to iloprost in mesenteric arteries from hypertensive rats (Liu et al., 2017). To establish the role of these receptors in the biphasic or monophasic response observed in male and female rats, respectively, vessels were preincubated in either EP_3_ receptor antagonist L‐798,106 (300 nmol·L^−1^) or IP receptor antagonist CAY‐10441 (100 nmol·L^−1^).

**FIGURE 2:**
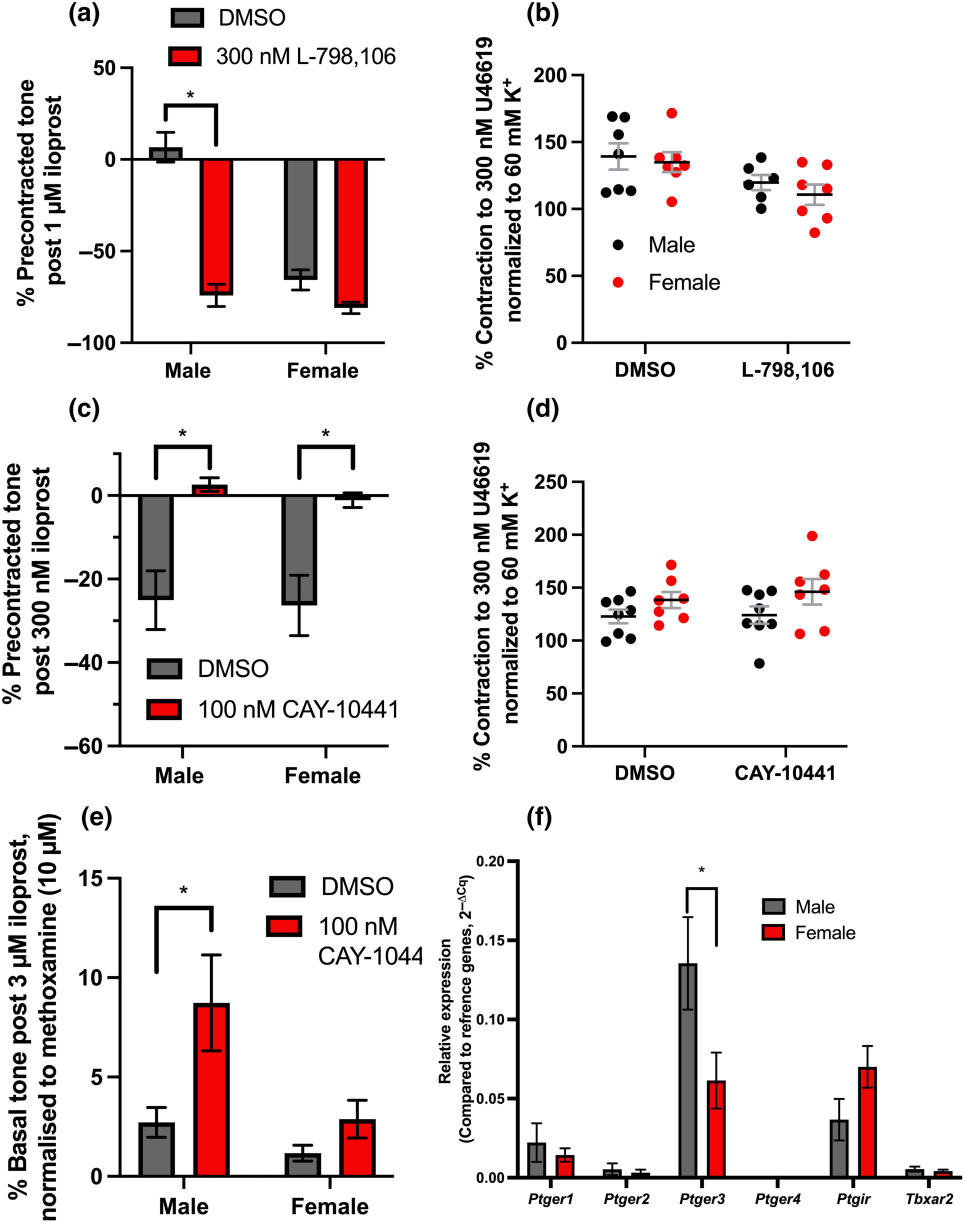
Iloprost‐mediated contraction and relaxation is blocked by EP_3_ and IP receptor‐mediated inhibition, respectively. Mean data for iloprost‐mediated vasoactive responses (a, 1 μmol·L^−1^; c, 300 nmol·L^−1^) within precontracted (300 nmol·L^−1^ U46619) mesenteric arteries from male and female rats preincubated in either solvent control (DMSO; a,c; *n* = 6–8), 300 nmol·L^−1^ L‐798,106 (a; *n* = 6–8) or 100 nmol·L^−1^ CAY‐10441 (c; *n* = 6–8). Scatter graph showing individual and mean values of stable precontracted tone from male and female arteries prior to adding iloprost to the chamber (b,d). Mean data for vasoconstriction from base line tension in response to 3 μmol·L^−1^ iloprost in male (black; *n* = 7) and female (red; *n* = 10) mesenteric arteries in the presence of DMSO or 100 nmol·L^−1^ CAY‐10441 normalized to peak contraction in response to 10 μmol·L^−1^ methoxamine (e). Relative gene expression of prostanoid receptors (*Ptger1–4 =* EP_1–4_, *Ptgi* = IP, *Tbxar2* = TXA_2_) normalized to stable housekeeper genes (*Canx*, *Cyc1*) expressed as 2^−ΔCq^ from male (black; *n =* 5) and female (red; *n =* 6–10) whole mesenteric artery lysates (f). A two‐way statistical ANOVA with a post‐hoc Bonferroni test was used to generate significant values (^*^ *P* < 0.05). *n* = number of animals used

**FIGURE 3:**
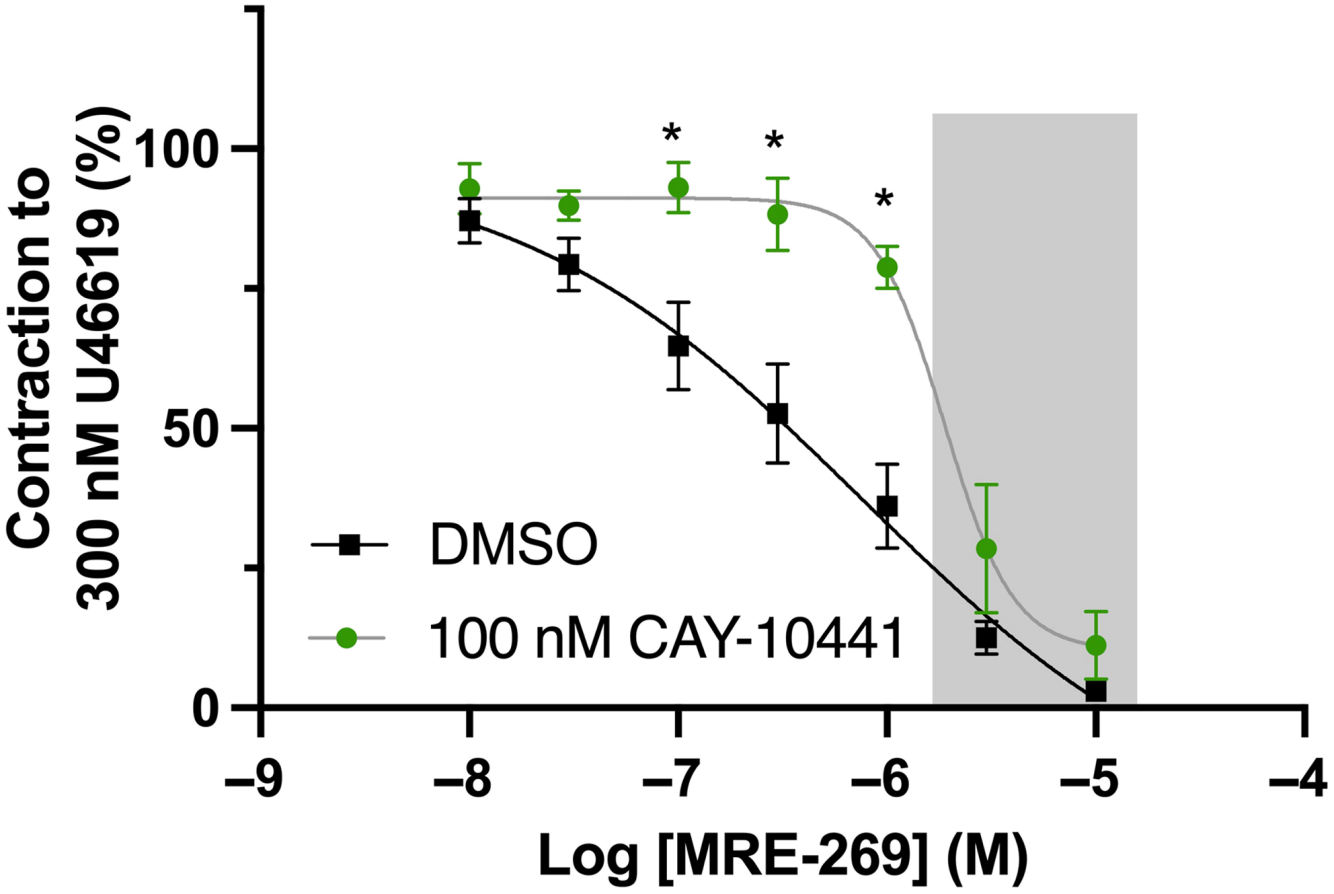
MRE‐269 relaxation is inhibited by CAY‐10441 to a threshold of 1 μmol·L^−1^ in male mesenteric arteries. Mean data of MRE‐269 (0.01–10 μmol·L^−1^) mediated relaxation of precontracted arterial tone (300 nmol·L^−1^) in vessels preincubated in DMSO solvent control (black; *n* = 7) or 100 nmol·L^−1^ CAY‐10441 (*n* = 5) in male mesenteric arteries. Grey box demonstrates non‐CAY‐10441 sensitive MRE‐269‐mediated relaxation. All values are expressed as mean ± SEM. A two‐way statistical ANOVA with a post‐hoc Bonferroni test was used to generate significant values (^*^ *P* < 0.05). *n* = number of animals used

In the presence of the EP_3_ receptor antagonist L‐798,106, 1 μmol·L^−1^ iloprost‐mediated contraction was converted to a relaxation (Figure 2a). In mesenteric arteries from females, EP_3_ receptor blockade had no effect (Figure 2a). In the presence of IP receptor antagonist CAY‐10441, 0.3 μmol·L^−1^ iloprost‐mediated relaxation was abolished in both groups (Figure 3a,b; *P* ≥ 0.05). Similarly, 3 μmol·L^−1^ iloprost evoked significantly greater contraction from base‐line tone in mesenteric arteries from male rats preincubated in CAY‐10441 when compared to mesenteric arteries from female rats (Figure 3e). No differences were observed in precontracted tone in vessels preincubated in either L‐798,106 or CAY‐10441 when compared to DMSO solvent control or between the sexes (Figure 2b/d). Subsequently, we performed quantitative PCR to determine the relative expression of prostanoid receptors (Table 1) in the mesenteric arteries from both sexes. Figure 2.F shows that expression of *Ptger2/4* (EP_2/4_) was negligible in mesenteric arteries from both sexes compared to *Ptger3* > *Ptgir* > *Ptger1* (EP_3_; IP; EP_1_), which were well expressed (Figure 2f). However, *Ptger3* was expressed at significantly lower level in mesenteric arteries from female rats compared to mesenteric arteries from males (Figure 2f). Thus, our data demonstrate that iloprost‐mediated relaxation in rat mesenteric arteries occurs predominantly via IP receptors, while contraction was caused by activation of EP_3_ receptors. Additionally, the absence of a biphasic response to iloprost in female mesenteric arteries was associated with a comparably smaller effect of EP_3_ receptor inhibition on iloprost‐mediated relaxation and a reduction in *Ptger3* expression.

### 3.2 Characterizing MRE‐269‐mediated relaxation

As iloprost has a plethora of potential targets, we used the clinically available IP receptor agonist, selexipag (NS‐304), to delineate the mechanisms underlying IP‐mediated relaxation. Application of selexipag produced concentration‐dependent relaxations of precontracted mesenteric arteries from male rats (Figure S3). However, this effect was insensitive to preincubation with IP receptor antagonist CAY‐10441 (Figure S3) thus, selexipag effects are non‐IP receptor dependent. It is now known that in the body, selexipag is metabolized into the active compound, inhibited by CAY‐10441 preincubation up to threshold of 1 μmol·L^−1^. At higher concentrations, the MRE‐269‐mediated relaxation was not sensitive to CAY‐10441 and therefore does not involve IP receptor activation. This non‐IP receptor‐mediated relaxation is highlighted by the grey box in Figure 3 and in the following investigations, MRE‐269 was used at concentrations ≤1 μmol·L^−1^ to ensure only IP receptor‐mediated effects were investigated.

### 3.3 A novel role for K_V_7.1 in shaping IP receptor‐selective agonists in mesenteric arteries

**FIGURE 4:**
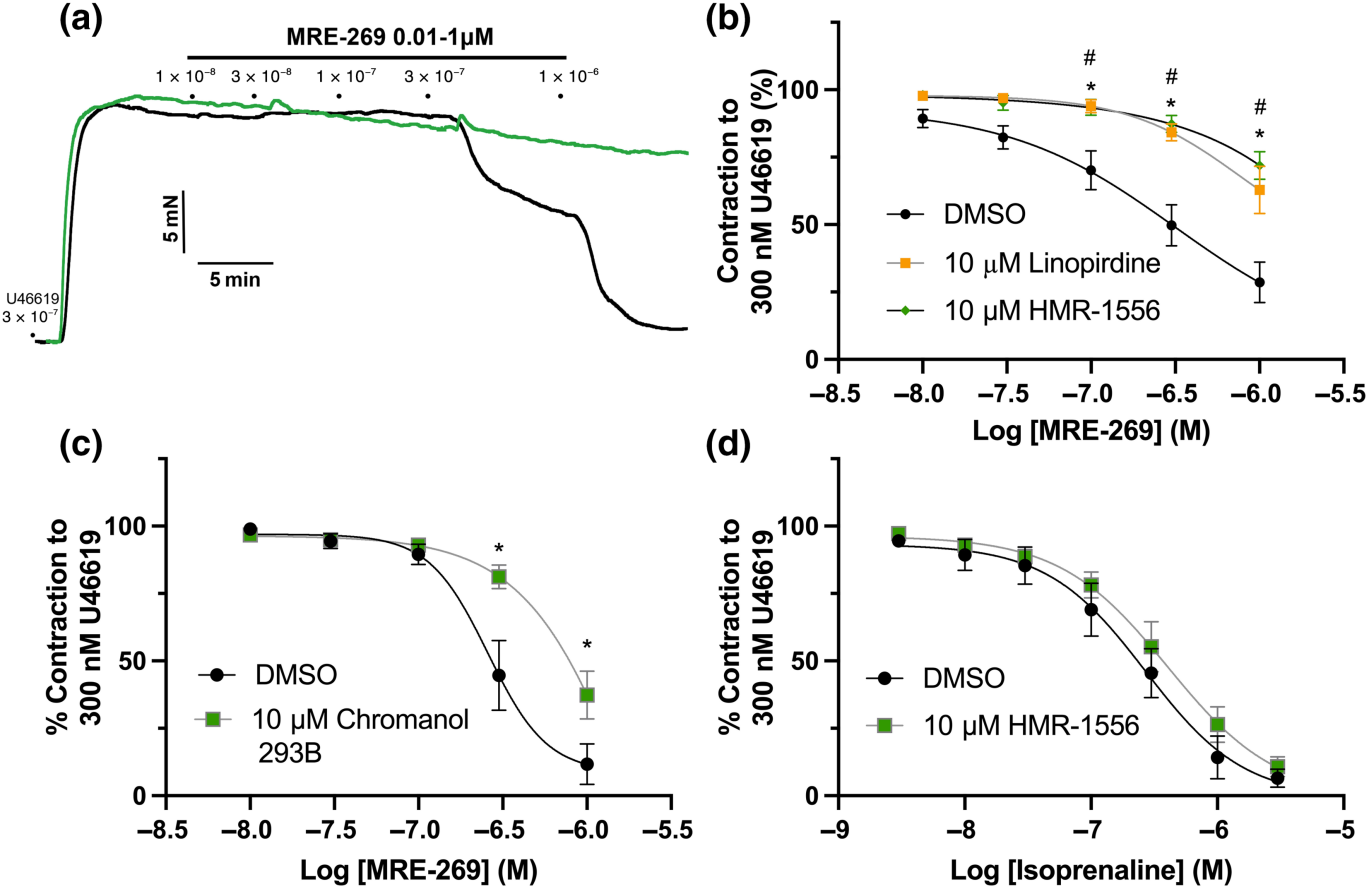
Linopirdine and HMR‐1556 attenuate MRE‐269‐mediated vasorelaxation in mesenteric arteries from male and female rats. Representative traces of MRE‐269‐mediated (0.01–1 μmol·L^−1^) relaxation of precontracted tone (300 nmol·L^−1^ U46619) within mesenteric arteries preincubated in either DMSO solvent control (a; black) or 10 μmol·L^−1^ K_V_7.1 specific blocker HMR‐1556 (a; green) from male Wistar rats. Mean data for MRE‐269‐mediated vasorelaxation (0.01–1 μmol·L^−1^) of precontracted tone (300 nmol·L^−1^ U46619) within mesenteric arteries preincubated in either DMSO (b; black) solvent control, 10 μmol·L^−1^ pan‐K_V_7 channel blocker linopirdine (b; yellow) or HMR‐1556 (b; green) or 10 μmol·L^−1^ K_V_7.1 seelective blocker Chromanol 293B (c; green; *n* = 7–10). Mean data for isoprenaline‐mediated relaxation in vessels preincubated in DMSO (black) or 10 μmol·L^−1^ HMR‐1556 (green) in male mesenteric arteries (d; *n* = 9). All values are expressed as mean ± SEM (a–f). A two‐way statistical ANOVA with a post‐hoc Dunnet (b) or Bonferroni (c,d) test was used to generate significant values (^*^ *P* < 0.05). *n* = number of animals used

Kv7 channels, especially Kv7.4 and Kv7.5, are functional endpoints for several G_s_‐linked receptors (see Barrese et al., 2020; Byron & Brueggemann, 2018). As such, we characterized the potential contribution of K_V_7 channels to IP‐receptor selective MRE‐269‐mediated relaxation. In mesenteric arteries from male rats, MRE‐269‐mediated relaxation was significantly attenuated by preincubation with the pan‐K_V_7 channel inhibitor linopirdine (10 μmol·L^−1^; yellow) when compared to DMSO (Figure 4, black). Strikingly, preincubation with the K_V_7.1 selective inhibitor HMR‐1556 (10 μmol·L^−1^) also inhibited MRE‐269‐induced relaxations to the same extent as linopirdine (Figure 4a,b, green;). The structurally dissimilar K_V_7.1 inhibitor chromanol 293B (3S,4R‐293B) also significantly attenuated MRE‐269‐mediated relaxation of male mesenteric arteries (Figure 4c). In contrast and consistent with previous reports (Stott et al., 2016), relaxations of mesenteric arteries induced by the mixed β‐adrenoceptor agonist isoprenaline were not affected by preincubation with 10 μmol·L^−1^ HMR‐1556 (Figure 4d).

**FIGURE 5:**
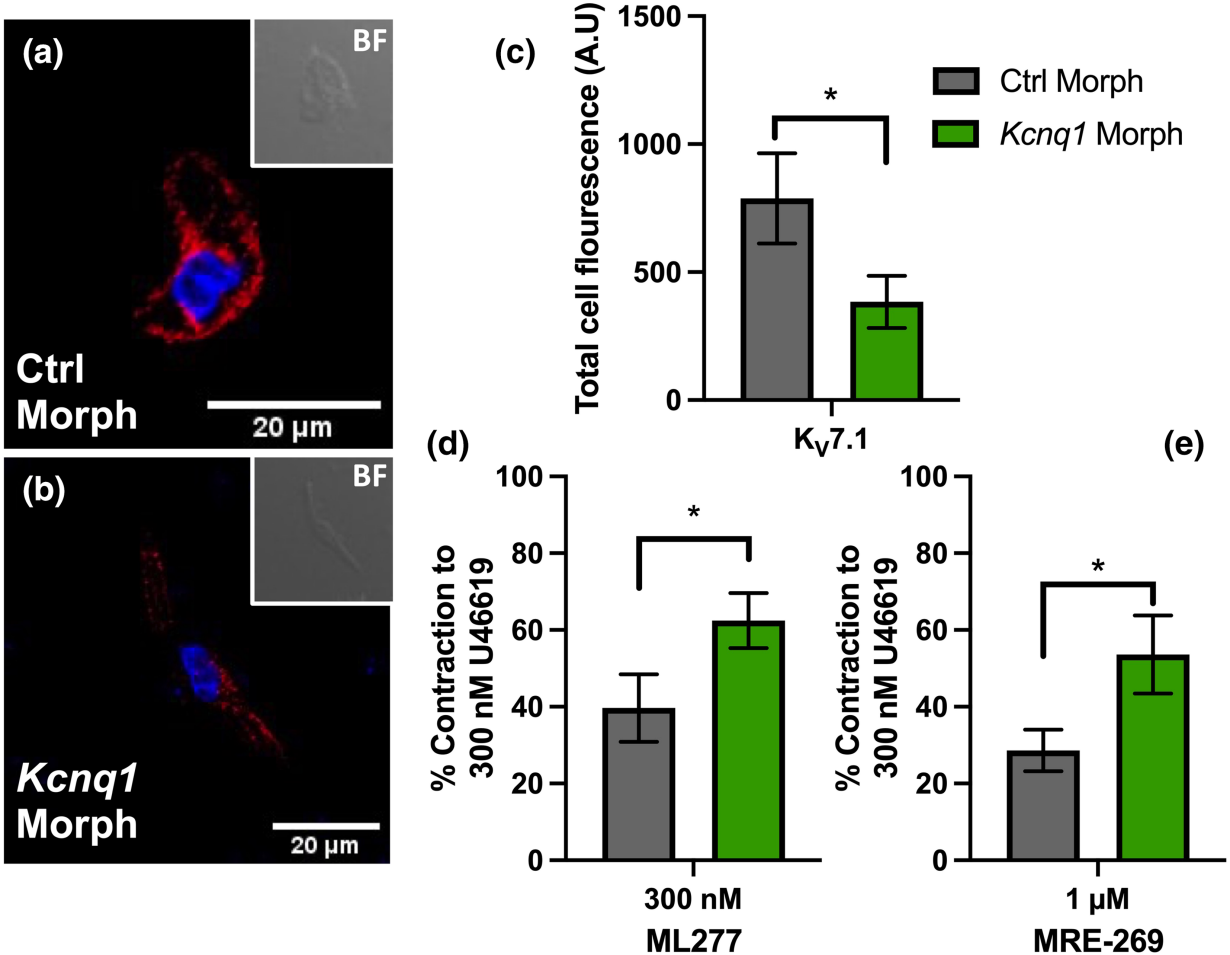
Molecular interference of *Kcnq1* via targeted morpholino knockdown impairs MRE‐269‐mediated relaxation. Representative immunofluorescence showing K_V_7.1 in isolated vascular smooth muscle cells from either scrambled control (Ctrl morph; a) or *Kcnq1* (*Kcnq1* morph; b) morpholino‐transfected mesenteric arteries. K_V_7.1 shown in red, nuclear staining in blue (DAPI [4′,6‐diamidino‐2‐phenylindole, dihydrochloride]). Insets show brightfield (BF) images of the cell. Mean data for total cell fluorescence measured in arbitrary units (A.U) for Ctrl morph (grey; *n* = 3; *N* = 10) and *Kcnq1* morph (green; *n* = 3; *N* = 10; d) transfected cells. Mean data for 300 nmol·L^−1^ ML277‐mediated relaxation (*n* = 7; e). Mean data for 1 μmol·L^−1^ MRE‐269‐mediated relaxation (*n* = 7; f). All values are expressed as mean ± SEM (a–f). A paired Student's *t*‐test was used to generate significant values (*P < 0.05). *n* = number of animals used, *N* = number of cells per biological repeat

To corroborate the contribution of K_V_7.1 to IP receptor‐mediated vasorelaxation, we transfected mesenteric arteries with morpholinos that prevent translation of Kv7.1 or a scrambled control. Immunocytochemistry with an antibody for K_V_7.1 validated by overexpression studies (Figure S1) showed a significant reduction in total cell fluorescence (A.U) in *Kcnq1* morpholino‐transfected arteries when compared to mismatch control (Figure 5a–c). Functionally, arteries incubated with mismatch control produced a significantly greater relaxant response to 300 nmol·L^−1^ k_v_ 7.1 activator ML277 (Baldwin et al., 2020; Yu et al., 2013) compared to *Kcnq1* morpholino‐transfected arteries (Figure 5d). Similarly, relaxation produced by 1 μmol·L^−1^ MRE‐269 was greater in arteries transfected with mismatch control morpholino when compared *Kcnq1* morpholino‐transfected arteries (Figure 5e). Thus, a reduction in K_V_7.1 protein was observed in conjunction with an attenuated relaxation by MRE‐269.

**FIGURE 6:**
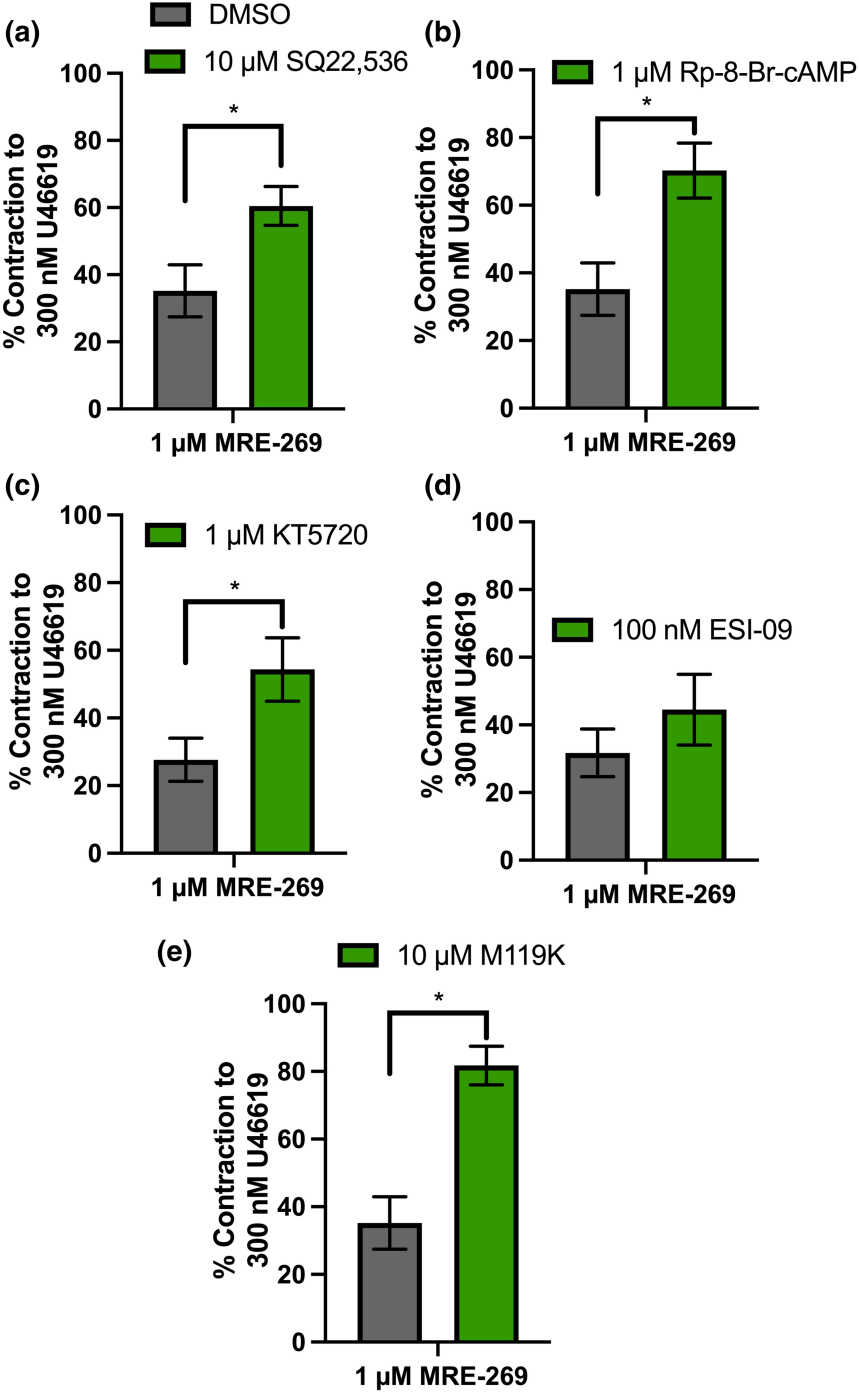
MRE‐268‐mediated relaxation is sensitive to adenylate cyclase, Gβγ and protein kinase A inhibition, but not effector protein activated by cAMP in male rat mesenteric arteries. Mean data for MRE‐269‐mediated vasorelaxation (1 μmol·L^−1^) of precontracted tone (300 nmol·L^−1^ U46619) within mesenteric arteries preincubated in either DMSO (a–e; *n* = 9; grey) solvent control or inhibitors of the following (green); adenylate cyclase–SQ22,562 (10 μmol·L^−1^; *n* = 7; a), protein kinase A–Rp‐8‐Br‐cAMP (1 μmol·L^−1^; *n* = 5; b)/KT5720 (1 μmol·L^−1^; *n* = 8; c), effector protein inhibited by cAMP–ES09 (100 nmol·L^−1^; *n* = 8; d) and Gβγ–ML119K (1 μmol·L^−1^; *n* = 7; e). All values are expressed as mean ± SEM (a–e). An unpaired Students *t*‐test was used to generate significant values (^*^ *P* < 0.05). *n* = number of animals used

Subsequently, we aimed to determine the signalling cascade activated in response to IP receptor stimulation. Previously, Schubert et al. (1996) demonstrated that iloprost‐evoked hyperpolarization of rat tail artery vascular smooth muscle cells is mediated via G_s_–cyclic adenosine 3′‐5′‐monophosphate (cAMP)–protein kinase A (PKA) stimulation of potassium currents (Schubert et al., 1996) in a process attributed to IP receptors, although not fully defined. Here, we similarly demonstrate that adenylate cyclase inhibitor SQ22,562 (10 μmol·L^−1^) and PKA inhibitors Rp8 (1 μmol·L^−1^) and KT5720 (1 μmol·L^−1^) significantly attenuated MRE‐269‐mediated relaxation (1 μmol·L^−1^; *P* ≥ 0.05; Figure 6a–c). In addition, inhibition of an alternative secondary signalling molecule activated by G_s_–cAMP, exchange protein directly activated by cAMP (EPAC), via ESI‐09 had no effect (100 nmol·L^−1^; Figure 6d). Finally, G_β**γ**_ inhibition by M119K (1 μmol·L^−1^) also significantly attenuated MRE‐269‐mediated relaxation (Figure 6e).

BK_Ca_ and K_ATP_ channels have also been identified as down‐stream targets of cAMP‐PKA‐dependent relaxations evoked by iloprost (Schubert et al., 1997). Here, we demonstrate that BK_Ca_ inhibitor iberiotoxin (100 nmol·L^−1^) but not K_ATP_ inhibitor glibenclamide (1 μmol·L^−1^) partially inhibited MRE‐269‐mediated relaxation in male mesenteric arteries (Figure S4A,B), though this failed to reach statistical significance.

### 3.4 Oestrus cycle‐dependent shifts in the sensitivity of MRE‐269‐mediated vasorelaxation to K_V_7 channel modulators

**FIGURE 7:**
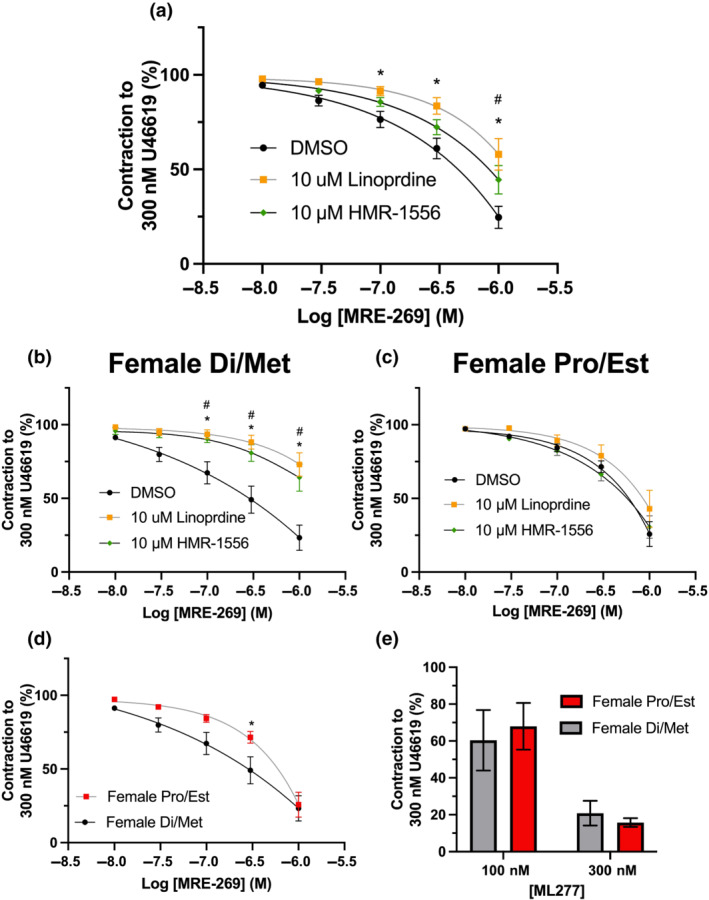
K_V_7 channel inhibition attenuates MRE‐269‐mediated vasorelaxation in mesenteric arteries from dioestrus/metestrus (Di/Met), but not proestrus/oestrus (Pro/Est) female rats. Mean data for MRE‐269‐mediated vasorelaxation (0.01–1 μmol·L^−1^) within mesenteric arteries preincubated in DMSO solvent control (a–c; black), 10 μmol·L^−1^ pan K_V_7 channel inhibitor linopirdine (a–c; yellow) or 10 μmol·L^−1^ K_V_7.1 specific inhibitor HMR‐1556 (a–c; green) from female Wistar rats. Data are expressed as either all female (a), female Di/Met (*n* = 5–7; b) and female Pro/Est (*n* = 6–8; c). MRE‐269 (0.01–1 μmol·L^−1^; d) and ML277 (0.01–0.03 μmol·L^−1^) mediated relaxation in vessels from either female Pro/Est (red; *n* = 6–8) or female Di/Met (black; *n* = 5–6). All values are expressed as mean ± SEM (a–e). A two‐way statistical ANOVA with a post‐hoc Dunnett's test (a–c) or Bonferroni test (d,e) was used to generate significant values (^*^ *P* < 0.05). *n* = number of animals used

The pan K_V_7 channel inhibitor linopirdine and K_V_7.1 channel inhibitor HMR‐1556 also significantly attenuated MRE‐269‐mediated relaxation in mesenteric arteries from female rats when compared to DMSO solvent control (Figure 7a;), though the latter to a smaller degree when compared to linopirdine. However, we observed two‐distinct populations of possible responses to MRE‐269‐mediated relaxation and its subsequent sensitivity to K_V_7 channel modulators, categorized into rats in dioestrus/metestrus or proestrus/oestrus (Pro/Est). Oestrus cycle stage was identified by defined histological changes in cells lifted from the cervix after killing (as per methods; Cora et al., 2015). Both the pan‐K_V_7 channel inhibitor linopirdine and K_V_7.1 selective inhibitor HMR‐1556 impaired MRE‐269‐mediated relaxation to the same degree in arteries from female in dioestrus/metestrus (Figure 7b) but had no effect in arteries from rats in proestrus/oestrus (Figure 7c). Moreover, MRE‐269‐mediated relaxation in arteries from female proestrus/oestrus rats was significantly less sensitive to the IP‐selective agonist than arteries from rats in dioestrus/metestrus (Figure 7d;). However, we observed no differences in K_V_7.1 activator ML277 (0.1–0.3 μmol·L^−1^) mediated relaxation on precontracted tone between the separated groups (Figure 7e). These data reveal an oestrus cycle stage‐dependent regulation of the contribution of K_V_7.1 to IP receptor‐mediated relaxation, which underpins diminished sensitivity to MRE‐269‐mediated relaxation during proestrus/oestrus.

## 4 DISCUSSION

To our knowledge, the present study is the first to highlight sex as a factor in the arterial response to prostacyclin mimetics. The study shows that application of iloprost to pre‐contacted mesenteric arteries from male rats produced bimodal responses, relaxation at low concentrations, followed by contraction at higher concentrations, whereas mesenteric arteries from female arteries presented with monophasic relaxation only. In arteries from both sexes the relaxant effect of iloprost was enhanced by EP_3_ receptor antagonist L‐798106 and was blocked by IP receptor antagonist CAY‐10441. Our data demonstrated iloprost‐mediated contractions in mesenteric arteries from male rats were more efficacious when compared to vessels from females, correlating with a higher level of *Ptger*_*3*_ expression. Finally, our data show that the selective IP receptor agonist MRE‐269 was a potent relaxant of precontracted mesenteric arteries from both sexes. This relaxation was inhibited by both the pan‐K_V_7 blocker linopirdine and K_V_7.1 selective blocker HMR‐1556 in arteries from males and dioestrus/metestrus females but strikingly not proestrus/oestrus rats. These findings are the first observation of K_V_7.1 as a downstream target of an endogenous vasoactive signalling cascade and reveal oestrus cycle‐dependent regulation of K_V_7 channels within the vasculature.

### 4.1 Iloprost‐evoked vasoconstriction

While principally regarded as a vasodilator, PGI_2_ mediates both relaxation and contraction of smooth muscle (Dusting et al., 1977; Liu et al., 2017; Moncada et al., 1976). PGI_2_ has subsequently been identified as an endothelial‐derived contracting factor produced in response to acetylcholine within rat aorta (Gluais et al., 2005), mesenteric (Liu et al., 2017), iliac (Zhang et al., 2021) and renal arteries (Zhang et al., 2021) in a process attributed to the activation of both EP and TP prostanoid receptors. Consistent with Liu et al. (2017), we show that high concentrations of iloprost‐evoked contractions were blocked by the EP_3_ receptor antagonist L‐798,106. As iloprost has a low affinity for TP receptors (Whittle et al., 2012), TP receptor knockout has no effect on PGI_2_‐mediated contraction in mesenteric arteries (Liu et al., 2017) and all vessels in this study were precontracted with U46619, a TP receptor agonist. TP receptors were not considered for the scope of this investigation. Additionally, EP_1_ receptor agonists do not elicit contractions in male mesenteric arteries (Kobayashi et al., 2011), and in agreement with previous findings (Kobayashi et al., 2011), a reduced expression of *Ptger1* was observed when compared to *Ptger3*. Furthermore, iloprost had negligible contractile effect in mesenteric arteries from female rats, which was associated with a lower expression level of *Ptger3* in these arteries.

### 4.2 K_V_7.1 underpins IP receptor‐mediated relaxation

Our data show that relaxations of mesenteric arteries mediated by low concentrations of iloprost were driven primarily through CAY‐10441‐sensitive IP receptor activation. We subsequently showed that CAY‐10441‐sensititve relaxations produced by the selective IP receptor agonist MRE‐269 were impaired by the selective K_V_7 channel blocker, linopirdine. Within the vasculature, of the five subtypes, *Kcnq4* > *Kcqn5 > Kcnq1* are the principally expressed transcripts with little to no expression of *Kcnq2/3* (Chadha, Zunke, Zhu, et al., 2012; Jepps et al., 2011; Yeung et al., 2007). K_V_7.4/K_V_7.5 alone however are implicated in the regulation of the resting membrane potential (Mackie et al., 2008) and basal tone (Mackie et al., 2008; Ng et al., 2011). In addition, pharmacological inhibition or molecular knockdown of K_V_7.4/7.5 impairs relaxations to many different relaxants including isoprenaline, calcitonin gene‐related peptide (CGRP), adenosine (G_s_ linked), atrial natriuretic peptide (ANP; cGMP linked) and adipose derived relaxant factors in several arteries (Byron & Brueggemann, 2018; Chadha et al., 2014; Gollasch, 2017; Khanamiri et al., 2013; Morales‐Cano et al., 2015; Stott et al., 2016; Stott, Barrese, et al., 2015). Our data suggest that IP receptor activation in male mesenteric arteries involves another GPCR that also relies on K_V_7 channels for functional responses. In contrast to previous reports (Lombard et al., 1999; Schubert et al., 1997) IP receptor‐mediated relaxation was not affected by K_ATP_ nor BK_Ca_ blockade. However, this discrepancy is potentially accounted for by a difference in vascular model used, as Lombard et al. (1999) investigated the rat middle cerebral artery and Schubert et al. (1997) used the rat tail artery.

Surprisingly, MRE‐269‐evoked CAY‐10441‐sensitive relaxations in mesenteric arteries from male rats were also potently inhibited by two structurally dissimilar K_V_7.1 selective inhibitors (HMR‐1556, chromanol 293B) and molecular knockdown of the channel. In contrast to K_V_7.4/K_V_7.5, the role of K_V_7.1 within the vasculature remains enigmatic. Though K_V_7.1 is expressed within vascular smooth muscle cells (Baldwin et al., 2020; Chadha, Zunke, Zhu, et al., 2012; Tsvetkov et al., 2017) and K_V_7.1 selective activators RL‐1 and ML277 are effective relaxants of precontracted arterial tone (Baldwin et al., 2020; Chadha, Zunke, Davis, et al., 2012), K_V_7.1 has not been identified as the downstream target of any endogenous vasoactive signalling cascades (Chadha et al., 2014; Stott et al., 2016; Stott, Barrese, et al., 2015). Yet in the present study, HMR‐1556 produced as full an inhibition as linopirdine, which suggests K_V_7.1, and not K_V_7.4/7.5, contributes to MRE‐269‐mediated relaxations. Under the same conditions, the mixed β‐adrenoceptor agonist isoprenaline produced relaxations that were not HMR1556 sensitive. Thus, our findings appear not to be an off‐target effect of HMR‐1556. Moreover, a role for K_V_7.1 in MRE‐269‐mediated relaxation was substantiated by morpholino‐induced reduction in K_V_7.1 protein levels. While further work is required to validate these findings, to our knowledge, the first to describe an effect on vascular reactivity by K_V_7.1 inhibition is our data. Additionally, our data support the notion that IP receptor‐mediated responses are cAMP‐PKA mediated. In agreement with previous work done by our lab (Stott et al., 2016, 2018; Stott, Povstyan, et al., 2015), we demonstrate that relaxations that are mediated PKA, but not EPAC, are also sensitive to G_β**γ**_ inhibition. The identification of G_β**γ**_ contribution to IP receptor‐mediated relaxation adds new complexity to the vascular response and gives credence to the novel role of G_β**γ**_ in the functional relationship between GPCRs and K_V_7s.

To date, comparably little is known as to how K_V_7 channels operate within the female. However, K_V_7 has been shown to regulate both human and murine myometrium (McCallum et al., 2009) and human chorionic plate artery (Mills et al., 2015) contractility. Intriguingly, while MRE‐269‐mediated relaxation was attenuated by linopirdine and HMR‐1556 in arteries from female rats, the effect of the latter was far smaller than in the male. When separated into oestrus cycle stages, arteries from females in dioestrus/metestrus expressed sensitivities to HMR‐1556 and linopirdine equivalent to the male, whereas arteries from females in proestrus/oestrus were entirely insensitive to either. However, K_V_7.1 activator‐ML277‐mediated relaxation was insensitive to changes in the oestrus cycle. As the functional output of pharmacological activation of the channel remains the same, the data indicate an oestrus cycle‐dependent impairment of K_V_7.1 channel coupling to IP receptor‐mediated relaxation. Oestrus cycle‐dependent regulation of vascular reactivity is a known but incompletely understood phenomenon (Jaimes et al., 2019) largely attributed to endogenous sex‐hormones, primarily, 17‐β oestradiol. 17‐β oestradiol negatively regulates K_V_7.1 in distal colic crypt cells and cardiac myocytes (Alzamora et al., 2011; O'Mahony et al., 2007; Rapetti‐Mauss et al., 2013; Waldegger et al., 1996). As previous work demonstrates that within the Wistar rat, oestradiol peaks within pro‐oestrus rats followed by comparably little to none in oestrus, metestrus and dioestrus (Nilsson et al., 2015), we propose that during pro‐oestrus, oestradiol levels rise, impairing K_V_7.1 coupling to IP receptor during proestrus/oestrus phase, thus reducing the potency of MRE‐269‐mediated relaxation and its HMR‐1556/linopirdine sensitivity, which does not recover until dioestrus/metestrus. Our data imply an oestrus cycle.

### 4.3 Perspectives

Sexual dimorphisms in cardiovascular physiology and pathophysiology are known (Pabbidi et al., 2018), whereby women express a cardioprotective factor or factors that differentiates the aetiology of vascular disease between age‐matched men and women. Chronic aldosterone treatment in male Wistar–Kyoto rats induces hypertension through endothelial dysfunction attributed to upregulated COX‐2 production of PGI_2_ (Blanco‐Rivero et al., 2005). Diminished EP_3_‐mediated contraction/expression in female rats may potentially negate the pathophysiological levels of PGI_2_ production observed by Blanco‐Rivero et al. (2005), contributing to the known cardioprotective phenotype expressed by females, though further work is required to validate this hypothesis.

## 5 CONCLUSION

The data of the present study demonstrate a remarkable sexual dimorphism in the vascular response to synthetic prostacyclin analogues and highlight the importance of considering sex as a determinant in vascular physiology. Strikingly, the potent relaxations to the selective IP receptor agonist MRE‐269 were sensitive to the Kv7.1‐selective blocker HMR1556 in mesenteric arteries from males and females in dioestrus/metestrus but not at all in proestrus/oestrus. These novel findings demonstrate both a functional role for K_V_7.1 in receptor mediated vascular reponses and cyclical changes in K_V_7 channel activity across the oestrus cycle, and form the basis of future investigations.

## AUTHOR CONTRIBUTIONS

S.N.B designed and implemented all experiments. S.N.B, E.A.F and L. M performed experiments, generated and analysed data. S.N.B and I.A.G drafted the manuscript. I.A.G oversaw the project and prepared the submission of the paper. I.A.G provided funding.

## CONFLICT OF INTEREST

The authors declare no conflict of interest.

## DECLARATION OF TRANSPARENCY AND SCIENTIFIC RIGOUR

This Declaration acknowledges that this paper adheres to the principles for transparent reporting and scientific rigour of preclinical research as stated in the *BJP* guidelines for Design & Analysis, Immunoblotting and Immunochemistry and Animal Experimentation, and as recommended by funding agencies, publishers and other organizations engaged with supporting research.

## Supporting information


**Figure S1.** Representative images of immunocytochemistry of CHO cells demonstrates anti‐body specificity. Chinese hamster ovarian (CHO) cells transfected with *Kcnq1* containing plasmids (A)*,* but not non‐transfected CHO cells, present with diffuse labelling of K_V_7.1, as seen in red (B). Insets contain 4′,6‐diamidino‐2‐phenylindole (DAPI) staining only, blue. Bar = 50 μm.

**Figure S2.** Cumulative concentration effect curves generated from iloprost mediated vasoactive responses. Mean data for iloprost mediated vasoactive responses (A; 0.001–1 μmol‐L^−1^) within pre‐contracted (300 nmol‐L^−1^ U46619) mesenteric arteries from male (black) and female (red) and female Wistar rats (A). The effect of equivalent cumulatively increasing volumes of DMSO solvent controls on pre‐contracted vessels from male and female Wistars (B). Iloprost mediated responses in vessels from male and female Wistars pre‐incubated in either solvent control (DMSO; C‐F; *n* = 6–8), 300 nmol‐L^−1^ L‐798,106 (C,D; *n* = 6–8) or 100 nmol‐L^−1^ CAY‐10441 (E,F; *n* = 6–8). All values are expressed as mean ± SEM (A‐F). A two‐way statistical ANOVA with a post‐hoc Bonferroni test was used to generate significant values (* P < .05). *n*; number of animals used.

**Figure S3.** Selexipag mediated relaxation was unaffected by CAY‐10441 in male mesenteric arteries. Mean data selexipag (B; 0.03–3 μmol‐L^−1^) mediated relaxation in vessels pre‐incubated in DMSO solvent control (black; *n* = 6) or 100 nmol‐L^−1^ CAY‐10441 (*n* = 5) in male mesenteric arteries. All values are expressed as mean ± SEM. A two‐way statistical ANOVA with a post‐hoc Bonferroni test was used to generate significant values (*P < .05). *n;* number of animals used.

**Figure S4.** MRE‐269 mediated relaxation is not attenuated K_ATP_ and BK_Ca_ inhibition. Mean data for MRE‐269 (A,B,C 0.01–10 μmol‐L^−1^) mediated relaxation in vessels pre‐incubated in DMSO solvent control (A,B,C; black; *n* = 4), 100 nmol‐L^−1^ BK_Ca_ channel inhibitor Iberiotoxin (A; blue; *n* = 5) or 1 μmol‐L^−1^ K_ATP_ channel inhibitor glibenclamide (B; blue; *n* = 5) in male mesenteric arteries. All values are expressed as mean ± SEM (A‐F). A two‐way statistical ANOVA with a post‐hoc Bonferroni test was used to generate significant values (* P < .05). *n*; number of animals used.

## Data Availability

The data generated herein are available upon reasonable request to the corresponding author.
