# Supplementary figures and images for: Sexual dimorphism in prostacyclin‐mimetic responses within rat mesenteric arteries: A novel role for KV7.1 in shaping IP receptor‐mediated relaxation
Source: Br J Pharmacol. 2022 Jan 21;179(7):1338–52. doi: 10.1111/bph.15722 (PMC9340493; doi:10.1111/bph.15722)

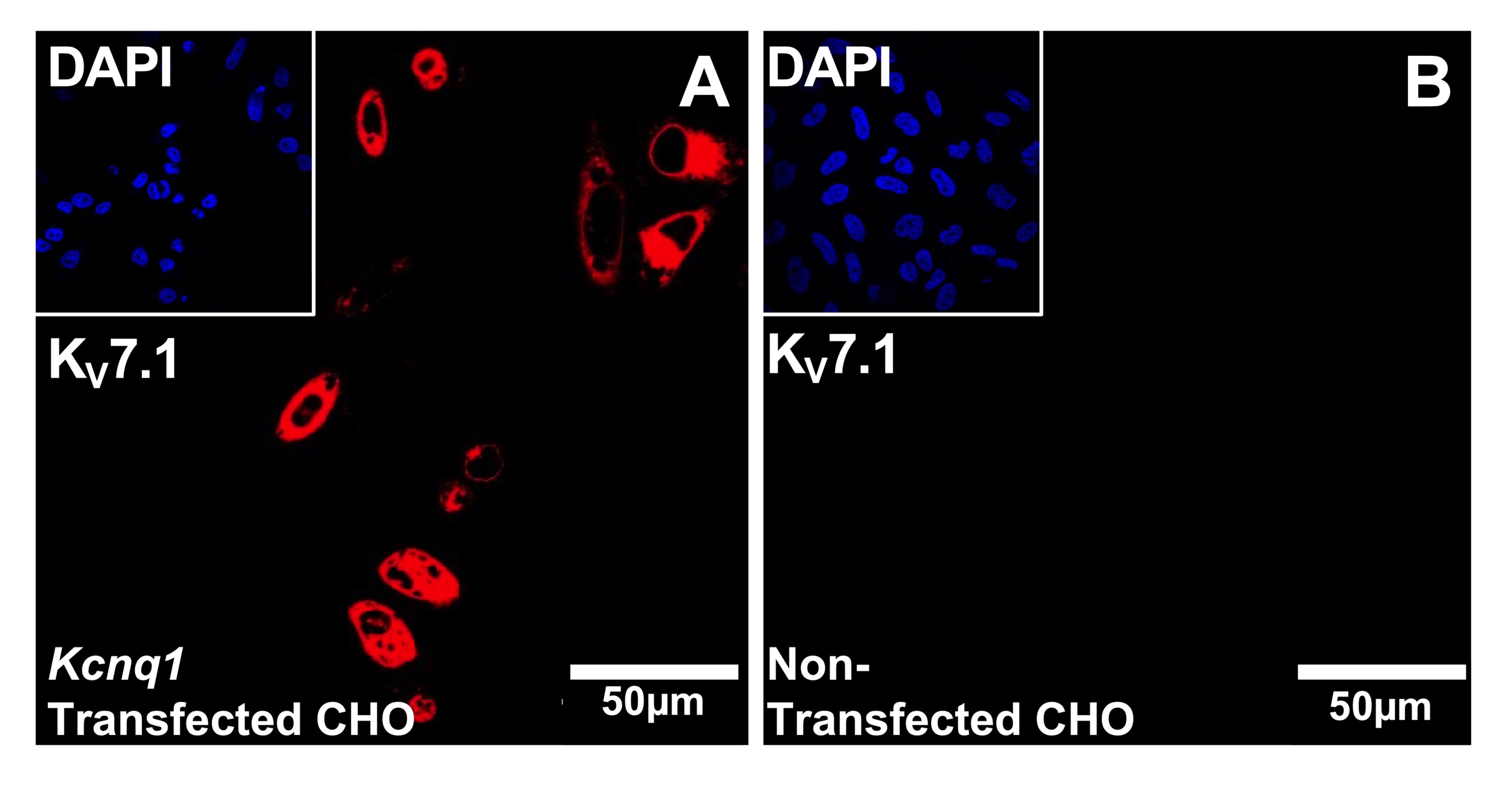

Supplement: Supplementary file 1 — Figure S1. Representative images of immunocytochemistry of CHO cells demonstrates anti‐body specificity. Chinese hamster ovarian (CHO) cells transfected with Kcnq1 containing plasmids (A), but not non‐transfected CHO cells, present with diffuse labelling of KV7.1, as seen in red (B). Insets contain 4′,6‐diamidino‐2‐phenylindole (DAPI) staining only, blue. Bar = 50 μm. [file BPH-179-1338-s004.jpg]

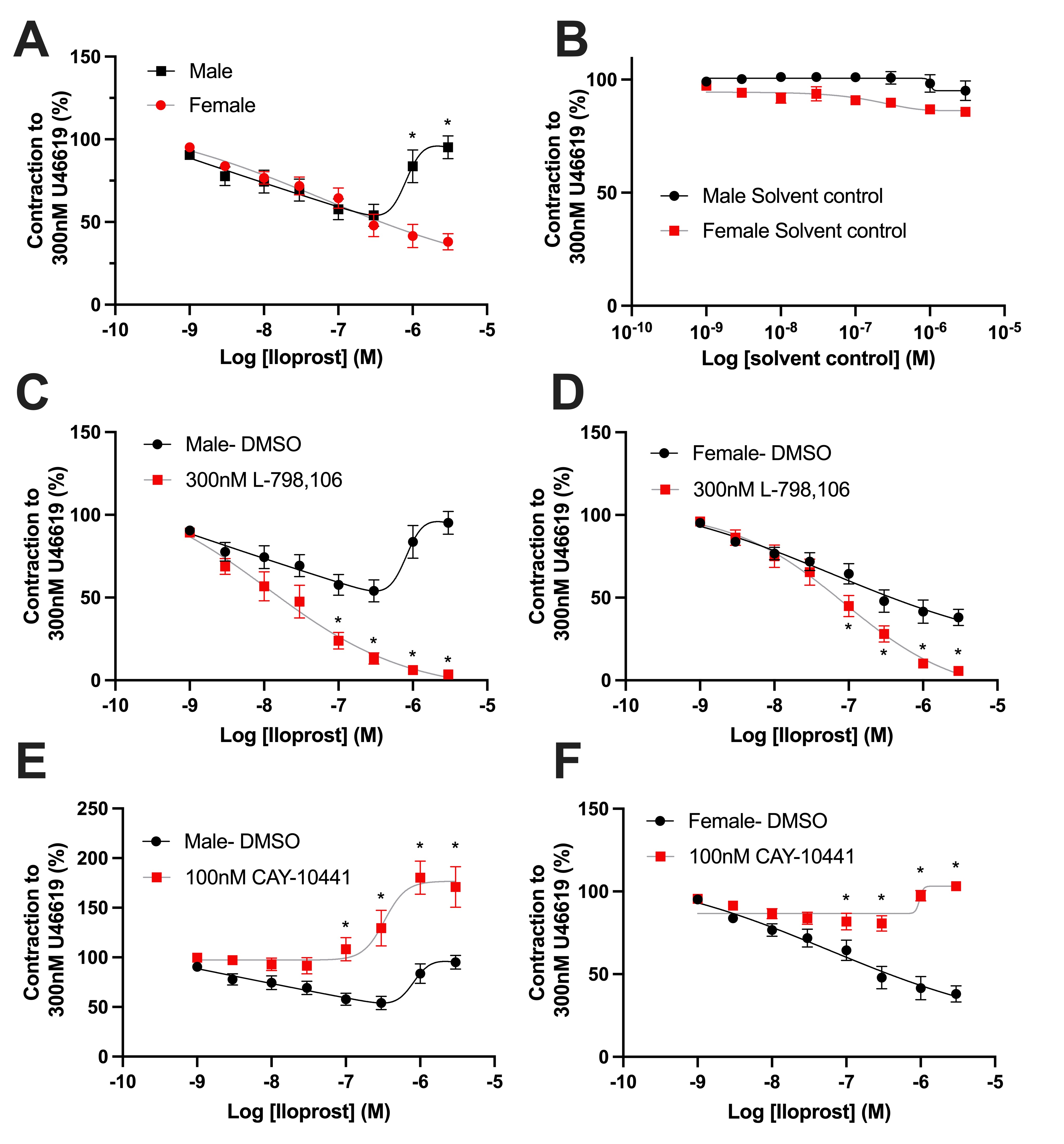

Supplement: Supplementary file 2 — Figure S2. Cumulative concentration effect curves generated from iloprost mediated vasoactive responses. Mean data for iloprost mediated vasoactive responses (A; 0.001–1 μmol‐L−1) within pre‐contracted (300 nmol‐L−1 U46619) mesenteric arteries from male (black) and female (red) and female Wistar rats (A). The effect of equivalent cumulatively increasing volumes of DMSO solvent controls on pre‐contracted vessels from male and female Wistars (B). Iloprost mediated responses in vessels from male and female Wistars pre‐incubated in either solvent control (DMSO; C‐F; n = 6–8), 300 nmol‐L−1 L‐798,106 (C,D; n = 6–8) or 100 nmol‐L−1 CAY‐10441 (E,F; n = 6–8). All values are expressed as mean ± SEM (A‐F). A two‐way statistical ANOVA with a post‐hoc Bonferroni test was used to generate significant values (* P < .05). n; number of animals used. [file BPH-179-1338-s003.jpg]

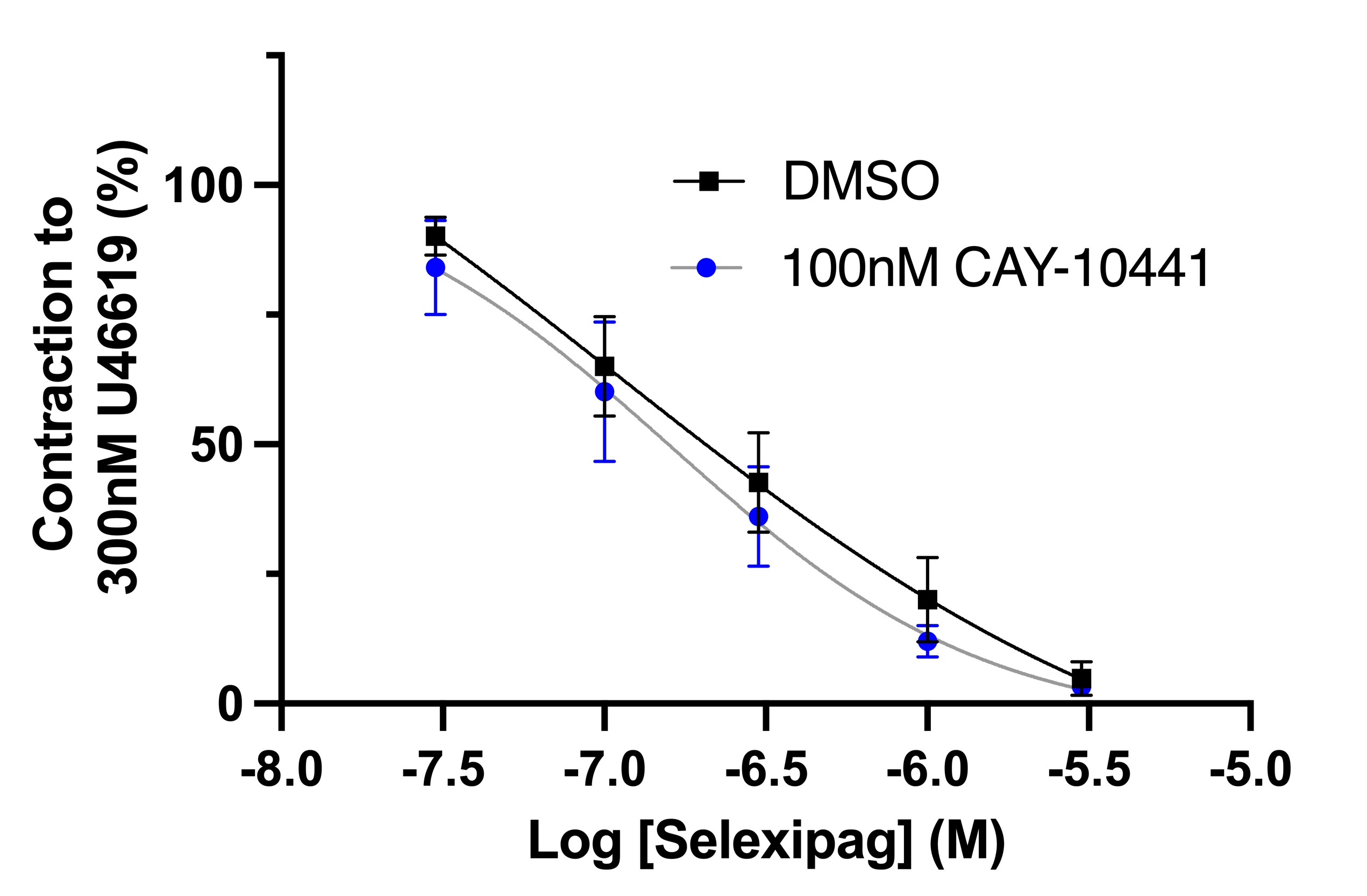

Supplement: Supplementary file 3 — Figure S3. Selexipag mediated relaxation was unaffected by CAY‐10441 in male mesenteric arteries. Mean data selexipag (B; 0.03–3 μmol‐L−1) mediated relaxation in vessels pre‐incubated in DMSO solvent control (black; n = 6) or 100 nmol‐L−1 CAY‐10441 (n = 5) in male mesenteric arteries. All values are expressed as mean ± SEM. A two‐way statistical ANOVA with a post‐hoc Bonferroni test was used to generate significant values (*P < .05). n; number of animals used. [file BPH-179-1338-s002.jpg]

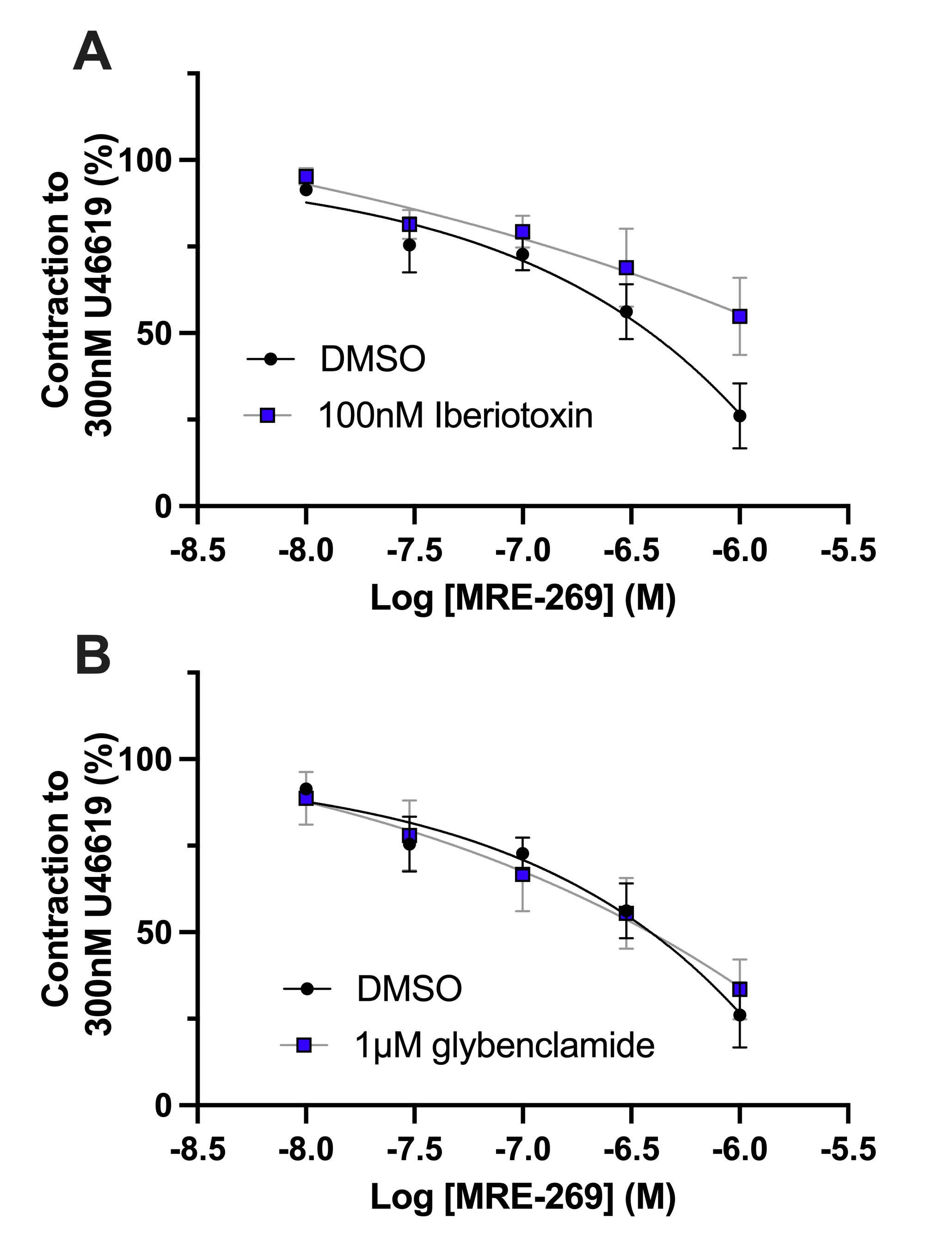

Supplement: Supplementary file 4 — Figure S4. MRE‐269 mediated relaxation is not attenuated KATP and BKCa inhibition. Mean data for MRE‐269 (A,B,C 0.01–10 μmol‐L−1) mediated relaxation in vessels pre‐incubated in DMSO solvent control (A,B,C; black; n = 4), 100 nmol‐L−1 BKCa channel inhibitor Iberiotoxin (A; blue; n = 5) or 1 μmol‐L−1 KATP channel inhibitor glibenclamide (B; blue; n = 5) in male mesenteric arteries. All values are expressed as mean ± SEM (A‐F). A two‐way statistical ANOVA with a post‐hoc Bonferroni test was used to generate significant values (* P < .05). n; number of animals used. [file BPH-179-1338-s001.jpg]
